# A qualitative examination of affect and ideology within mass media interventions to increase HIV testing with gay men garnered from a systematic review

**DOI:** 10.1111/bjhp.12461

**Published:** 2020-07-31

**Authors:** Darren Langdridge, Paul Flowers, Julie Riddell, Nicola Boydell, Gemma Teal, Nicky Coia, Lisa McDaid

**Affiliations:** 1 School of Psychology and Counselling, The Open University, UK; 2 School of Psychology & Health, University of Strathclyde, Glasgow, UK; 3 MRC/CSO Social and Public Health Sciences Unit, University of Glasgow, Glasgow, Scotland; 4 Usher Institute of Population Health Sciences and Informatics, University of Edinburgh, Edinburgh, Scotland; 5 Institute of Design Innovation, Glasgow School of Art, Glasgow, Scotland; 6 NHS Greater Glasgow and Clyde, Glasgow, Scotland; 7 Institute for Social Science Research, The University of Queensland, Brisbane, Australia

## Abstract

**Objectives:**

Increasing appropriate HIV testing among men who have sex with men (MSM) is crucial to HIV prevention. Mass media interventions are effective in promoting testing, but to date, there has been little examination of their active content.

**Design:**

We conducted a qualitative analysis of intervention materials (n = 69) derived from a systematic review of mass media interventions designed to improve testing with MSM.

**Methods:**

Visual data were analysed for their affective and ideological content using a novel method drawing on concepts from semiotics (i.e., broadly speaking, the analysis of signs).

**Results:**

Whilst affect was not explicitly theorized or examined in any of the studies, there are clearly identifiable affective elements implicitly at play in these interventions. Four thematic categories of affect/ideology were identified including (1) sexual desire and the ‘pornographication’ of the gay/bisexual male subject; (2) narratives of romance and love; (3) fear, threat, and regret; and (4) ‘flattened’ affect.

**Conclusions:**

This is the first study to examine and detail the affective and ideological aspects of intervention content in this field. Using analytic techniques such as those reported here, in addition to approaches that focus on the manner in which intervention content address more proximal determinants of behaviour, can provide a rich and potentially more useful evidence base to assist with future interventions.

HIV testing remains a critical step in both providing treatment and reducing infection rates. Undiagnosed HIV infection remains the primary source of continuing ongoing transmission (Skarbinski et al., 2015). Recent figures suggest there are around 4,200 men who have sex with men (MSM) in the United Kingdom who are currently undiagnosed (Nash et al., 2018). It is vital therefore that we ensure that MSM are tested regularly. Mass media interventions designed to increase testing are one means to facilitate this aim. The primary aim of this paper was to generate a greater understanding of recent HIV testing intervention content – in terms of its affective and ideological content – to both advance intervention theory and enable academics and policymakers to design and commission better interventions for the future. To this end, we present a qualitative examination of visual materials from mass media interventions designed to increase HIV testing, garnered from a systematic review.

Understanding the affective content of mass media health interventions is important if we are to better engage and motivate recipients of these communications (Cameron & Chan, 2008; Williams & Cameron, 2009). There has been a growth of interest in affect within health psychology (DeSteno, Gross, & Kubzansky, 2013), notably through the way it has been incorporated within self-regulation models of behaviour (see Cameron & Leventhal, 2003, for a useful review of this approach). Most work has focused either on affect as part of a positive/negative emotional response within a broader theoretical model (e.g., Scheier & Carver, 2003) or specifically on fear and threat as a means to motivate behavioural change (Cameron & Chan,2008; Myrick, 2015; Ruiter, Kessels, Peters, & Kok, 2014). Key to much of this work and the present study is a recognition of how affect serves to mobilize engagement with media interventions and also potentially facilitate behaviour change. Emotional arousal serves to produce a readiness to act (Frijda, 1986), and affective information may also play a critical role in judgement and decision-making (Finucane, Alhakami, Slovic & Johnson, 2000).

Examining the ideological underpinning of affective content is similarly critical when it comes to how we might engage and motivate recipients of health interventions to change behaviour. Ideology – the taken-for-granted (naturalized) meaning underpinning a set of beliefs – is a key structuring factor of affect (Fox, 2015; Myrick, 2015; Wetherell, 2012) and therefore also a key structuring factor for individual emotional responses to health interventions. We argue here that an analysis of mass media behaviour change interventions benefits from being understood beyond a focus on individual feeling states alone, in large part because the point of mass media is to reach a population rather than individual. That is, we contend – and intend to show in our analysis – that consideration of the way that our emotional life is patterned by broader social and cultural forces is critical if we are to understand how we might best engage our target audience and motivate them into action through particular health communications. The ideological underpinning will facilitate or limit viewer identification (Igartua & Barrios, 2012; Igartua & Casanova, 2016; Murphy, Frank, Chatterjee, & Baezconde-Garbanati, 2013) and particularly the possibility of narrative transportation in which a person becomes immersed in a story (Green & Brock, 2000), and it is critical when designing interventions to ensure that the underpinning belief framework is therefore concordant with the ideological positioning of the intended audience.

Visual images are commonly used in public health interventions and represent an important route for engaging and motivating viewers (Cameron & Chan, 2008; Williams & Cameron, 2009). Image-based material is processed more quickly and at greater depth than linguistic or verbal information and is more easily recalled (Cameron & Chan, 2008). Visual images also provoke more immediate and powerful emotional responses, at least in part likely due to the way that perception of images appears to be neurologically closely related to concrete experiential processes themselves (Cameron & Chan, 2008). Furthermore, research across a range of domains provides empirical support for the value of using imagery in public health interventions (see, e.g., Cameron & Williams, 2015; Noar, Hall, et al. 2016; Rus & Cameron, 2016). The fact that images are likely to be the element recalled most readily from a intervention and as such act to anchor further health intervention representations (Cameron & Leventhal, 2003) is a powerful reason for ensuring that image selection is explicitly included as part of the intervention design process and grounded in the best available evidence (Cameron & Chan, 2008; Williams & Cameron, 2009).

The creation of public health mass media interventions involves myriad decisions, some explicit and many implicit, about the messages we are trying to convey and means by which we engage and motivate the targeted population. In other words, when designing interventions, we seek to ‘encode’ (Hall, 1980) particular content in order to engage and motivate ourtarget audience to effect some behavioural change, and affect is a vital – albeit often neglected – element in achieving that end (Cameron & Chan, 2008; Langdridge, Davis, et al., 2019; Myrick, 2015; Tannenbaum et al., 2015). Much of this process is intuitive rather than explicitly reasoned (Green & Witte, 2006) and is a complex and hitherto relatively unexplored aspect of the intervention design process (although see Murray et al., 2016). Whilst it is rarely possible to audit or analyse the actual decisions made by design teams, or the content of actual commissioning briefs, it is possible to retrospectively analyse intervention content. In this way, an analysis of the way that affect (and ideology) are encoded in existing public health materials is a valuable start to developing a more sophisticated understanding of recent practice, in the service of ultimately understanding what works, and what we might do differently in the creation of future interventions.

The broader systematic review from which the data for this study have been garnered has sought to utilize a pluralistic approach to better understand intervention content and its relationship to effectiveness. To date, we have examined effectiveness per se (McDaid et al., 2019), behaviour change techniques and theoretical domains (Flowers et al., 2016), and the social marketing, mode of delivery, and use of imagery focusing upon the social position of the viewer and context of viewing (Riddell et al., under review). In contrast to these analyses, the focus in the present study is on an analysis of the cultural determinants of visual materials, as visual materials provide one of the primary means for the encoding of affect within mass media oriented health communications (Langdridge, Davis, et al., 2019; Noar, Francis, et al., 2016). The analysis includes a consideration of the affective nature of visual materials garnered from a systematic review of HIV testing interventions, along with consideration of the ideological apparatus that structures the particular presentation of materials in this behavioural domain. Affective (and ideological) material is, however, not easily categorized or counted, as remarked upon by Williams and Cameron (2009) and so requires methods beyond those that might be normative within more cognitive models of health psychological research. As such, we aim to demonstrate the value of a relatively new approach to making sense of the affective and ideological nature of visual health intervention materials using techniques derived from semiotics.

## Method

### Systematic review

Details of the systematic review are reported in McDaid et al., 2019); however, we have included a summary of the procedure undertaken for the review in order to provide a background to the current analysis.

#### Search strategy

Five electronic databases were searched for studies published since 2010, using detailed search strategies and standard MESH terms for HIV, MSM, and social marketing/mass media interventions. References were checked and included as appropriate.

#### Study selection

**Figure 1:**
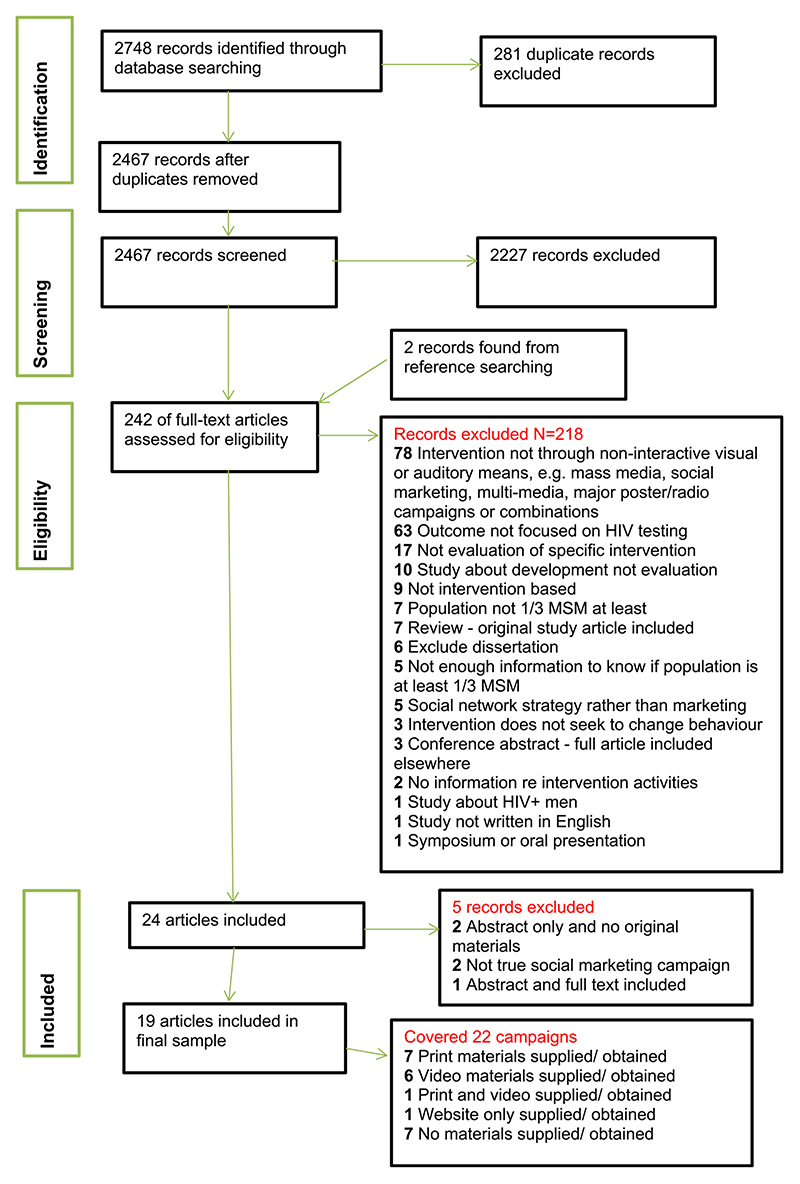
PRISMA flow chart. [Colour figure can be viewed at wileyonlinelibrary.com]

Studies published prior to 2010 and with intervention materials in languages other than English, Spanish, or Italian were excluded. Studies in which MSM constituted at least one third of the sample, included interventions that sought to change behaviour through non-interactive, visual or auditory means, and included HIV testing as an outcome were included. The procedure for identifying relevant papers is shown in the PRISMA diagram in Figure 1.

#### Data extraction

The systematic review resulted in 19 studies reflecting 22 interventions for analysis. Visual materials were requested from authors as part of the systematic review process and also searched for within related materials. The criteria for inclusion were kept broad to include all posters and leaflets, along with video materials, even those that were composed of text alone. A maximum of three requests were made to study authors for intervention materials. In the original analysis, this resulted in visual materials for 14 interventions with 70 individual items (McDaid et al., 2019). In the present study, conducted some months later, there were some minor differences in the materials available for analysis. Three videos were not available for analysis due to dead links (Chiasson et al., 2009; Hirshfield et al., 2012), only two still images found instead. This resulted in the set of materials listed in Table 1, a total of 69 separate items for 14 interventions that were then subject to analysis.

### Analysis of affect and ideology

Building on our previous work (Langdridge, Davis, et al., 2019), the analysis of health communication materials herein draws directly from the work of the semiotic theorist Roland Barthes (1977, 2009). The work of Barthes, and semiotics in general, is in large part concerned with the study of signs and how we make sense of them (Chandler, 2017). We are not conducting a traditional formalized (social) semiotic analysis here but rather are conducting a psychologically informed discursive analysis drawing on a number of key concepts from Barthes (1977, 2009) to critically interrogate the visual materials derived from systematic review. Our primary focus is on the affective content of these materials, their ability to engage the viewer, and the ideological apparatus that structures the imagery and that opens up and closes down particular ways of reading it. The analysis was conducted by the first author (DL) with the entire process checked by PF. In addition, all paper authors checked the coding and analysis, offering comment and critique until an agreed upon analysis was formulated.

The analytic procedure was as follows:

1. The collated visual materials were systematically examined for affect, drawing on principles from the work of Barthes (1977, 2009). This involves multiple parsing of the data, beginning with an analysis of explicit (literal) mentions of affect (examination of denotation) followed by examination of implicit cultural meanings (examination of connotation), value judgements, and associations. This includes consideration of the use of metonymic signs (where they represent something else, e.g., an image of a cockerel representing a penis) and synecdochical signs (where part of a sign represents a bigger whole, e.g., a watercooler as symbolic of office romance).
2. The detailed analysis of affect was followed by a critical examination of ideology in order to deepen the connotative analysis, the analysis of the cultural determinants of the affective content. This required critical reflexive investigation into the ideological positions (and particular subjects, e.g., sexualized gay man vs. heteronormative man in a faithful dyadic relationship) brought into play through the use of visual imagery and associated text. The aim here was to grasp the social effects of meaning within the materials being analysed and how this might be read by the viewer. Following Barthes (1977, 2009), this included consideration of how the (invariably implicit) ideology might serve to affectively engage the target audience (or not) whether through identification, transportation, or some other form of emotional power.
3. The last stage involves bringing together key concepts and ideas from the stages above through a thematic analysis such that this material is brought together into coherent themes. This way of bringing otherwise disparate elements together allows for the reader to gain insight into the key overarching elements implicated in the analysis of affect and ideology, and how this might impact on the effectiveness of the communication in engaging the viewer and mobilizing them into action. Key here is a process of coding moving from purely descriptive codes to more interpretive analysis identifying patterns in the data (Miles & Huberman, 1994). See Table 2 for further detail on the coding process that underpins this analysis.

The identification of connotation and ideology within visual imagery is complex and not easily systematized. Van Dijk (1998) argues that ideological content has the quality of’I know it when I see it’ because the analysis is, in large part, an analysis of one’s own culture – and awareness of other cultures – in relation to the material being subject to analysis. It requires the analyst to critically engage the materials themselves and also their own subjectivity, adopting a strong reflexive stance (see Langdridge, Davis, et al., 2019). There are some simple albeit rather basic ways to identify ideology, such as the use of discourse involving ‘us vs. them’ or ‘ingroup vs. outgroup’ membership (Van Dijk, 1998), but ultimately judgements must be determined on the basis of producing an account that is persuasive to one’s peers. The form of analysis described herein must be subject to juridical account by an informed audience through an open and auditable process (Ricoeur, 1970, 1976, 1981). In the first instance, this is within the research team, as seen here, and then finally through the review process and among the wider body of expert academic peers.

## Findings

The data analysis focused on identifying patterns of affect and ideology resulted in four thematic categories. The first was concerned with sexual desire and the potential ‘pornographication’ of the gay/bisexual male subject. This thematic category represented the dominant position with respect to studies included in this study. The second theme involved material deploying narratives of love and romance. The third used more common empirically driven affects within health psychology of fear, threat, and regret. Finally, the fourth thematic category represented visual material in which there appeared to be no engagement with affect at all or – more accurately – material working with some sort of flattened affect.

### Sexual desire and the ‘pornographication’ of the gay/bisexual male subject

**Figure 2:**
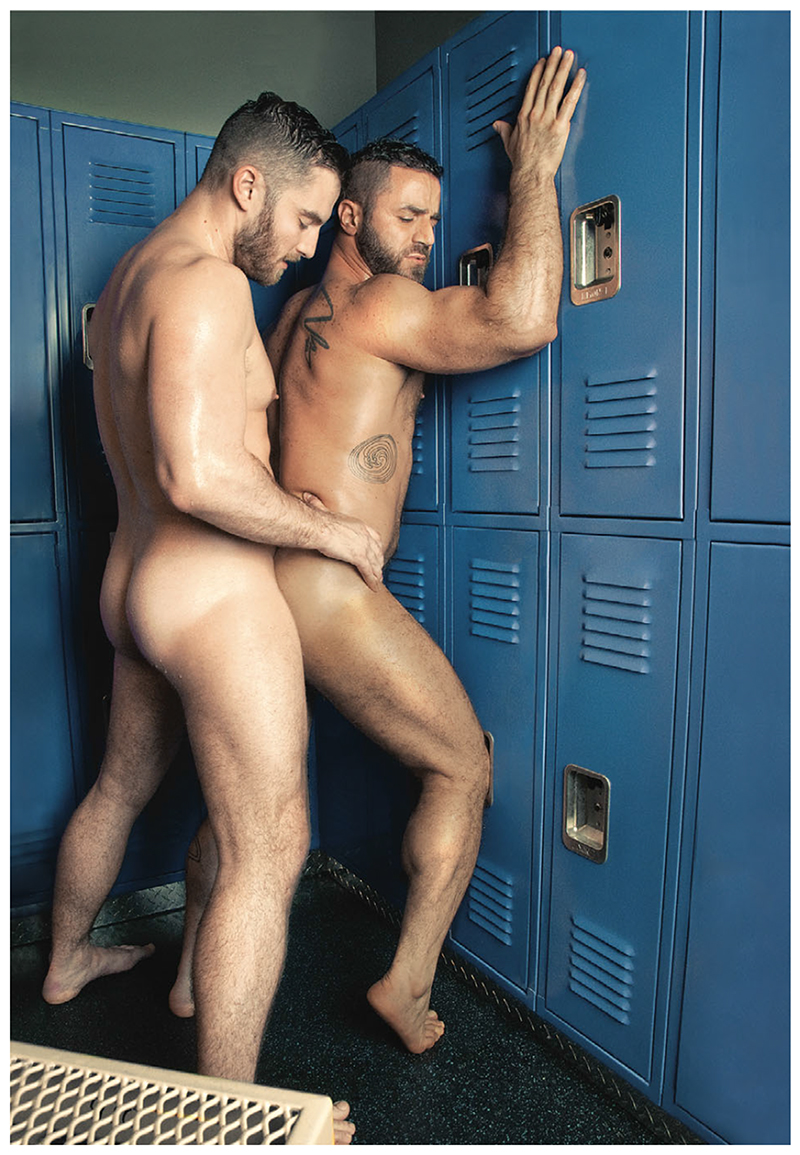
Example image (poster 3) from Gilbert et al. (2013). [Colour figure can be viewed at wile yonlinelibrary.com]

The most significant finding is the dominance of a focus, at least in the majority of Western studies (n = 7), on sexual desire as the primary means of engaging the viewer (see Figure 2). That is, the majority of the studies reviewed did not deploy emotions such as fear or threat known to influence behavioural change at all but instead employed a gay/bisexual male pornographic aesthetic to ‘entice’ the viewer (see Table 2). They are in many ways rather one dimensional with only the occasional use of humour deployed to off set the pornographic aesthetic and seductive style. Here, we can see the ‘pornographication’ of health communications (McNair, 1996; see also Tyler & Quek, 2016), in which there is the incorporation of pornographic imagery into mainstream public health messages. More specifically, it could be argued that this is a classic exemplar of ‘porno-chic’ (McNair, 2002: 61), where we see:

[T]he representation of porn in non-pornographic art and culture; the pastiche and parody of, the homage to and investigation of porn; the post-modern transformation of porn into mainstream cultural artefact for a variety of purposes.

The purpose here is complex for the way that this ‘porno-chic’ serves to deploy a familiar aesthetic for the particular gay/bisexual male target audience whilst also operating in an educative manner. The separation of ‘actual’ pornography from ‘porno-chic’ is somewhat arbitrary and arguably problematic (Tyler & Quek, 2016) but here appears relatively clear cut, with these intervention materials notably devoid of the presence of an ‘actual’, rather than implied, penis.

Within the images deploying the general porno-chic aesthetic the penis is everywhere and yet also nowhere (cf. Flowers, Langdridge, et al., 2013). That is, there is an implicit suggestion of a barely hidden or even erect penis but with penetration implied only, never shown, and intimacy intimated but ultimately only acted out. This is a – very soft – soft pornography indeed, arguably more about the borrowing of a familiar aesthetic tope than the sexual arousal of the viewer. The images might seduce or titillate to some extent but are rather tame by contemporary standards, especially given the proliferation of such imagery in public settings (McNair, 1996, 2002), and the ubiquitous availability of hardcore pornography online.

Even so, comedy is utilized in a number of interventions, as if to ameliorate the impact of the pornographic aesthetic further, to temper the suggestion of sexual arousal in the viewer. Comedy is commonly used as a trope to lessen the impact of the sexual, especially where it is thought this might be deemed challenging (Baker, 2005; Langdridge & Butt, 2004; Wilkinson, 2009). Innuendo is used here in many of these images with ostensibly non-sexual objects standing in for the penis: the cockerel features as ‘the cock’, for instance. Humour is also used to mobilize the viewer beyond a purely voyeuristic relationship to the imagery. For instance, the inclusion of animals against an attractive naked torso in the ‘Drama downunder’ materials work to produce a visual formulation of an affectively powerful phrase, in a quirky manner but one that is consistent with the production of humour (see, e.g., Warren & McGraw, 2016). A kitten and frightened facial expression serve to visualize the ‘scaredy-cat’, a goldfish that there is ‘something fishy’ going on down below, whilst a cockatoo serves to represent the ubiquitous ‘cock’ that is centre stage in all these interventions.

The male figures at the visual heart of these sexual desire-driven interventions subscribe to contemporary norms of male beauty and are well-muscled, with any body hair neatly trimmed. They represent the quintessential ‘ideal type’ gay/bisexual man (Lanzieri & Hildebrandt, 2011), the perfectly built mesomorph, well-groomed, and universally desirable, at least within the visual sphere of contemporary male advertising (Patterson & Elliott, 2002). And just like the general advertising sphere, these men are not representative of the broader target audience (cf. Kolbe & Albanese, 1996). There is a lack of portrayal of ectomorphic (thin and lightly muscled) or endomorphic (soft and round) men, in spite of gay/bisexual cultures in which these body types are themselves desired (e.g., twink and bear). As a result, the images will likely not work through processes of identification as the majority of viewers will be unable to recognize themselves in these images (cf. Langdridge, Davis, et al., 2019). Instead, they must serve to seduce the viewer through presentation of an ‘ideal type’ and/or speak through a familiar visual language, the language of pornography, perhaps thereby offering up some sense of familiar pornographic transportation into a narrative of sexual desire (Green & Brock, 2000).

This imagery implies a particular contemporary form of hegemonic masculinity is at play in these communications. This is a well-groomed ‘new’ man whose body is his project (Giddens, 1991) and who is now both object of the gaze of the other as much as the one who gazes (Patterson & Elliott, 2002). There is some evidence from research on attractiveness that men often seek men who are physically similar to what the culture determines is masculine (Bailey, Kim, Hills & Linsenmeier, 1997) and here we see the representation of a gay/bisexual subcultural ‘porno-chic’ norm (Baker, 2005). This is a highly individualized sexual male who is confident about his own sexual desires and pleasures (Baker, 2005). The question is how well these interventions work for a viewer for whom the pornographic aesthetic is less familiar or less comfortable and for whom the notion of being highly sexually agentic is problematic?

### Narratives of romance and love

In stark contrast to the communications employing a pornographic aesthetic, a Chinese intervention (Tang et al., 2016) focused strongly on a narrative of love and romance. It was the only intervention focused on love and romance and was also notable for being produced through a crowdsourced competition designed to encourage a more ‘bottom-up’ approach to intervention design (Zhang et al., 2015). Here, the primary means of interpellation (Althusser, 1972) was through activating the subject with a sense of responsibility for the other (person and wider society) through the affective complex of love and care. This video-based intervention used a very traditional narrative of romance in which a chance meeting leads to a loving relationship. The characters here, like the ‘porno-chic’ interventions, can be thought of as stereotypically beautiful but much less sexual with no nudity or explicit reference to sexual acts. The focus here is clearly on the couple as active agent and not the singular individual, as is apparent within many of the other Western intervention materials. The key affective mobilization comes in the form of identification and narrative transportation allied to the wish to work together as a couple to protect each other and also an implied wider society. Responsibility figures centrally here with sexual desire and pleasure featured in the background only.

The narrative of romance portrayed in this film is powerful, with a strong story able to hook even the casual viewer. It is a classic love story. Identification is likely to be highly significant here for emotional engagement, predominantly in the form of identification with character and/or narrative (Baudry, 1970/2011; Green & Brock, 2000; Metz, 1977/1982). This is consistent with recent work on persuasive communication (e.g., Igartua & Barrios, 2012; Igartua & Casanova, 2016; Murphy, Frank, Chatterjee & Baezconde-Garbanati, 2013) which suggests that if we wish to engage the audience, then we either need to ensure that the material presented offers up the chance of character identification or presents a powerful narrative that speaks beyond personal character identification. This video offers up the possibility of both character identification and also transportation through a powerful narrative, albeit one that is deeply traditional in relationship form and style (the married dyadic couple).

**Figure 3:**
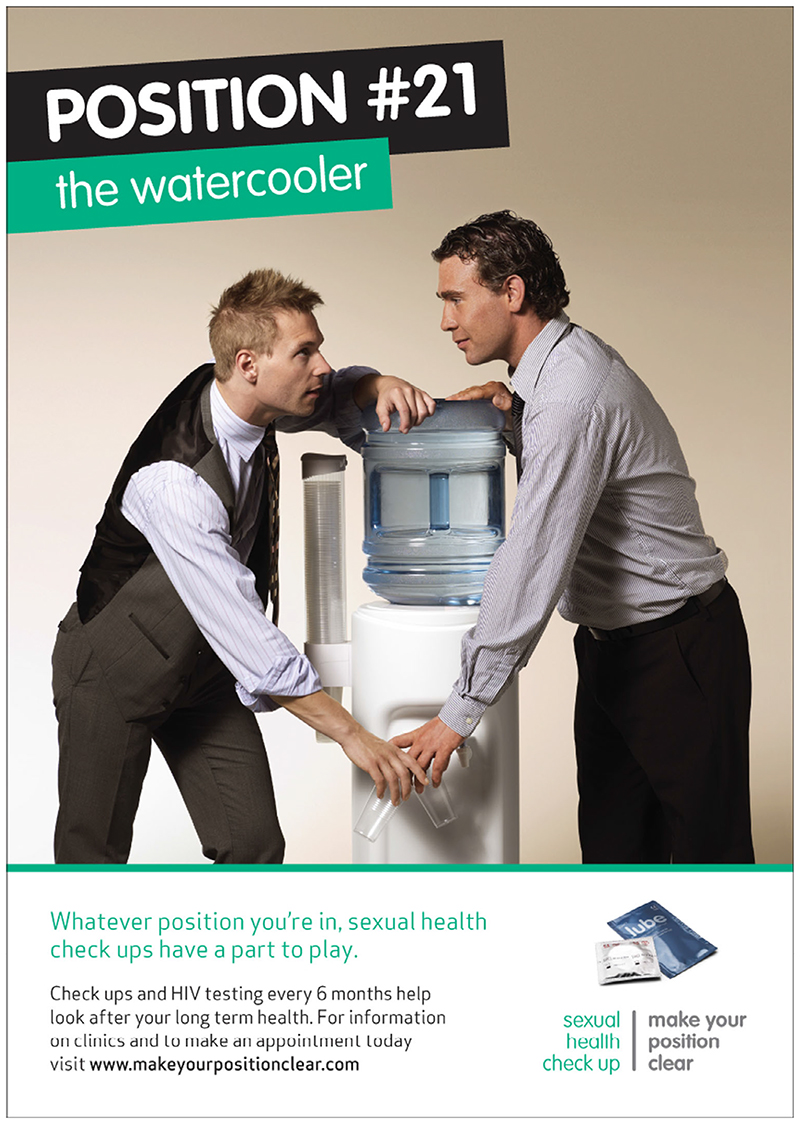
Image from Flowers, McDaid etal. (2013) ‘The Watercooler’. [Colour figure can be viewed at wileyonlinelibrary.com]

The UK ‘Make your position clear’ intervention (Flowers, McDaid, et al., 2013) is notable for the way that it combines the porn aesthetic described above with an underlying narrative of romance. The two men in this poster story are attractive and often naked but embedded within this intervention is a story of romance through the use of images of meeting at the ‘watercooler’ and supermarket (see Figure 3). The men also look directly at each other, apparently a longing look into each other’s eyes rather than at the viewer. This creates a sense of intimacy that is similar to that in Tang et al. (2016) albeit it one that is much more immediately and obviously sexual. This imagery contrasts strongly with the blank backgrounds in the same intervention’s posters of the men naked and engaged in (acted) sexual positions, which conjure up a more straightforwardly pornographic aesthetic. This intervention also embeds a sense of humour in the visual imagery through the use of sexual positions and associated nomenclature.

### Fear, threat, and regret

One intervention particularly engaged a more traditional affective focus on threat (Blas et al., 2010; Cameron & Chan, 2008), albeit even here this was more complex than simply about trying to scare the viewer into action. This intervention from Peru was targeted at a specific audience of young MSM and consisted of two videos driven by distinct narratives about potential concerns around testing positive for HIV. The aesthetic of the videos was uniform and mostly presented in documentary style with the central characters ‘realistic’ in that they did not appear to be particularly beautiful ‘model-type’ men. The videos included flashback sequences and overlaid internal voices from the characters expressing their thoughts and fears. They engaged directly with character-led fears about becoming HIV-positive, but all ended with some sense of relief, either through a person testing negative or by revealing that with treatment a HIV-positive diagnosis is not as problematic as it might appear. The characters include the following: (1) a young man who does not identify as gay but has sex with men; and (2) a gay/bisexual young man, and his friend who later discloses he is HIV-positive. Identification is the most likely means of engaging the viewer but whilst they work with a narrative format, they are not particularly affectively powerful. This is probably because they appear overly dramatized and rather long, to a Western viewer albeit potentially not to their intended local audience where such a format may be normative.

The health marketing video (Tang et al., 2016) involving a cartoon character being seduced is also noteworthy for how it engages with fear and regret to motivate behaviour. The primary character was shown to drop the umbrella (symbolizing a condom) and then to appear scared under dark clouds whilst searching for information online about HIV. They are then presented as happy and relieved when receiving a negative test result at a clinic. This cartoon video also uses humour, as mentioned above, to ameliorate the gentle sexual quality that is present. The tone here is only mildly sexual in the first instance, focused primarily on heterosexual desire and seduction rather than sex itself, but even so the comical cartoon tone is employed to further shift the focus away from the sexual content being implied.

### ‘Flattened’ affect

Finally, it is worth briefly mentioning three interventions in which there was an apparent attempt to ‘flatten’ the affect associated with HIV testing and focus more on a cognitively oriented information transmission approach, seemingly to normalize the process for the viewer of the material and remove any sense this activity might be threatening or otherwise emotionally challenging. That is, these interventions did not seek to affectively move people to test or engage affect to lessen the sense of threat implied but instead eschewed – whether deliberately or not – the use of affect entirely within their intervention materials. ‘Point of Care Testing’ (POCT) (West et al., 2015), ‘I’m testing’ (James, 2015), and ‘United against Aids’ (Prati et al., 2016) all sought, albeit in somewhat different ways to normalize HIV testing. In the process, however, there was not a uniform focus on gay, bisexual, and MSM as the target audience, which may reduce the power of these communications in terms of viewer identification, for example, through seduction, or transportation. POCT (West et al., 2015) offered up a simple documentary-style presentation designed to demystify the process of testing. For those already engaged with the idea, then it may prove helpful for alleviating concerns about the details of the process, but the patient was a middle-age woman rather than an obviously gay, bisexual, or MSM man. There was little here to draw the viewer in or otherwise engage someone who was not already motivated to engage with the material. The ‘I’m testing’ and ‘United against Aids’ interventions were undoubtedly high-quality professional interventions, with glossy materials that were stylishly conceived. Both presented male and female figures, albeit ‘I’m testing’ including a well-known gay British celebrity doctor among them. ‘I’m testing’ was focused on images of stereotypically beautiful young people, smiling set against bright colours. There was a sense of warmth and happiness in this intervention, apparently seeking to associate the process of HIV testing with positive affect. ‘United against Aids’ was a more diffuse intervention with a wide range of figures presented (different ages and sex) within a high-art setting. The imagery was aesthetically appealing but not obviously likely to be resonant to any particular viewer. There was a sense of people coming together to fight HIV/Aids, but it lacked a targeted message or audience focus.

### Effectiveness, affect, and ideology

The analysis of visual imagery was dependent on access to the intervention materials and, whilst a good body of materials was collated for analysis, it remains limited by having the materials for only 14 of the interventions, as well as by the differences in how effectiveness was measured across the studies. The effectiveness analysis revealed that all but four studies (Chiasson *et al*.; Guy *et al*.; Hirshfield *et al*.; Prati *et al*.) included in the analysis demonstrate effectiveness, in either HIV testing itself or the antecedents of HIV testing (e.g., knowledge). As such, it was not possible to discern any clear patterns linking affect and ideology with effectiveness within this data set. There is evidence suggesting that interventions across the range of affective/ideological themes may prove effective (in increasing uptake of testing/increasing knowledge) but without further information about the processes underpinning these outcomes, it is not possible to determine any associations.

## Discussion

To our knowledge, this is the first study, both globally and within the United Kingdom, to examine a coherent and rigorously identified set of HIV intervention materials from evaluated mass media interventions in order to systematically appraise how they reflect and utilize ideology and affect to elicit behaviour change among their intervention recipients. A range of approaches have been developed to examine and learn from the proximal determinants of behaviour change, for example, the behaviour change wheel (Michie et al., 2011), the theoretical domains framework (Atkins et al., 2017), form of delivery (Dombrowski et al., 2016). In contrast, far less has been concerned with understanding the distal – particularly cultural – determinants of behaviour change and how they are incorporated within intervention materials by accident or design. It is hoped the analysis offered here provides a novel and complementary perspective to extant approaches to understanding intervention content (Ogden, 2016), particularly in the context of growing interest in affect within health psychology.

We identified four distinct affective themes through the analysis reported here. These included ‘Sexual desire and the “pornographication” of the gay/bisexual male subject’; ‘Narratives of romance and love’; ‘Fear, threat and regret’; and ‘“Flattened” affect’. Our analysis asserts that affect plays a potentially important role in public health interventions concerned with HIV, albeit one that is rarely explicitly formulated or even acknowledged. As we have seen in similar work on antimicrobial resistance (Langdridge, Davis, et al., 2019), affect is a present but tacit aspect of mass media health communications concerned with HIV but remains undertheorized and relatively unexplored in the intervention design process according to the published materials. The exception to this perspective concerns those studies we have placed within the category of’flattened affect’, in which the focus is clearly on the presentation of factual information and communication of cognitive rather than affective material. The present study is the first study to show there may be value in exploring and testing the role of affect – and associated ideological discursive structures – in producing effective mass media health interventions designed to increase HIV testing, and likely other sexual health behaviours. This may range from the effective utilization of existing theory concerning affect, such as that concerned with threat (see Cameron & Chan, 2008; Myrick, 2015), to designing and testing domain-specific interventions using novel, and to date relatively untested, affects.

The primary affective theme was one focused on sexual desire, deploying a ‘pornochic’ aesthetic in the main. This also appeared to be relatively successful in terms of helping to produce effective behaviour change, although we must be cautious here not to overinterpret any association. However, the use of pornography – and therefore a porn aesthetic – sits uneasily with many (Corneau, Beaulieu-Prévost, Bernatchez & Beauchemin, 2017), with notions of guilt and exploitation potentially at play. In addition, this aesthetic trope relies on very particular body types and appearances, which may themselves alienate potential viewers and/or play a role in reifying a limited array of acceptable/desirable images of gay and bisexual men. There is arguably a need for balance between using a normalized gay/bi aesthetic (focused on ‘porno-chic’) to attract the viewer set against possibility of viewer identification, affective engagement beyond arousal, and indeed wider moral concerns about the proliferation of imagery that may itself contribute to body image concerns within the target population.

The theme focused on a pornographic aesthetic, all of which are within Western contexts, contrasts markedly with the narratives of love and responsibility and also comic cartoon seduction in the studies by Tang et al. (2016), which were based in China and the love and romance video also crowdsourced. Might there be more space for this aspect of the experience of gay/bisexual men (and MSM) in addition to desire alone or is this something uniquely appropriate to collectivist cultures? The study by Flowers, McDaid etal. (2013) bridges these two stances somewhat albeit still primarily with a pornographic aesthetic and has proven successful. Given the known value of identification and transportation as a means for engagement (and persuasion) (Baudry, 1970/2011; Green & Brock, 2000; Metz, 1977/1982), particularly with powerful narratives (Igartua & Barrios, 2012; Igartua & Casanova, 2016; Langdridge, Gabb & Lawson, 2019; Murphy, Frank, Chatterjee & Baezconde-Garbanati, 2013), it is rather surprising how rarely identification with characters and transportation through narrative seeking to look after the health and well-being of themselves and/or their partner/s is deployed in such interventions. That is, the studies drawing on a porno-chic aesthetic are highly individualistic in nature, with little consideration of the relational (and caring) context of the potential audience for the intervention or indeed the relational transmission of STIs such as HIV.

The use of threat, fear, and relief is fascinating for quite how explicitly they seek to affectively move the viewer and also more generally for how little use there is of this established affect within this particular behavioural domain (cf. Green & Witte, 2006). Whilst the study by Blas *et al*. is focused on the Peruvian context where sexual health matters may be quite distinct from Western societies, there is a powerful attempt to ‘move’ the viewer through the affective quality of the videos. Here is the most obvious, perhaps too obvious, intervention in which established modes of health communications affects are deployed. The use of affect, and particularly fear and threat, is proven in many behavioural domains (see Cameron & Chan, 2008 and Myrick, 2015, for useful summaries) and here too appears to be relatively effective. This intervention is more nuanced in that it offers something beyond mere threat and is also striking for the contrast with more usual strategies being deployed within the other more sex-saturated interventions designed to increase HIV testing deployed in the West.

Arguably, there is scope for interventions that seek to bring the three distinct domains of desire, romance, and threat together, accepting the use of fear and threat as psychological motivators remains controversial with HIV interventions (Green & Witte, 2006). The mostly one-dimensional nature of the porn aesthetic desire-based interventions is striking, with enormous scope for this to be expanded to incorporate other affects that are already established within the health psychology literature (such as threat), as well as affects beyond the individual (such as care/love for another). By contrast, the non-Western interventions we identified through our search strategy are striking for how they move beyond the highly individualized nature of the sexual subject that forms the basis of most other interventions whilst at the same time eschewing a porn aesthetic. Whilst the cultural context may be a factor in the success (or not) of different (individualistic vs. collectivist) strategies, it may also be the case that these decisions are being based on the ideologies of the researchers and/or those commissioning the work, alongside key contextual factors such as where such images are being deployed (see Riddell et al., under review, for more on this), rather than empirical evidence concerning the effectiveness of particular affects within particular cultural contexts.

### Limitations

Finally, it is important to reflect on the novel method used here, its strengths and weaknesses, the limitations of the study and any lessons learned. This study, like the previous study using this method (Langdridge et al., 2019), is innovative in how it highlights hitherto relatively unexamined, and undertheorized, elements within public health interventions. It provides valuable new information to help inform future intervention development and testing. There remains considerable work to do, however, as the analysis is necessarily limited by the availability of data for analysis within a systematic review process. The sole focus of the systematic review, from which data for this paper were drawn, was on HIV testing and only studies that included HIV testing as an outcome were included. Interventions that sought to influence HIV transmission risk behaviours or HIV-related stigma more broadly were beyond the scope of this study, but could also impact on HIV testing behaviours. Additionally, mass media interventions may be developed and delivered by community-based organizations, without formal evaluations published in academic journals. Their exclusion from this review is a limitation, especially given there may be considerable innovation within these materials. Finally, it is important to note that most of the included studies were conducted prior to the introduction or availability of pre-exposure prophylaxis (PrEP) for HIV prevention (the use of antiretroviral HIV treatments by HIV-negative individuals to prevent the acquisition of HIV). PrEP has dramatically altered the context of HIV testing within HIV prevention, but could not be reflected on consistently within this review and will need to be considered within any future intervention development.

The study is also limited through an analysis that is distanced from the original intended context of the intervention, with images subject to critical analysis outside of their original viewing context (see Riddell et al., under review, for more on this). Also, ideally, it would be possible to map this analysis of affect and ideology to effectiveness but that was not possible in this study. The majority of studies included in this analysis demonstrated some level of effectiveness with more subtle distinctions in effect not available for analysis. Future work on intervention development might address this issue through inclusion of an explicit plan for the use of affect/ideology and its specific evaluation.

This study has not only sought to provide new information about the nature of the key affective components within the specific behavioural domain of HIV testing among gay/bisexual and men who have sex with men, but also further detailed a method that has previously only been used within one other behavioural domain (antimicrobial resistance). In this study, we have sought to more clearly demonstrate the coding process such that other researchers may be better able to conduct a similar analysis. This attempt at beginning the process of codifying this method is of course necessarily imperfect given the nature of the method and reliance on a critical reflexive stance among the researchers. This is not a method that – at this stage, at least – can be conducted in a reliably systematic manner by anyone who wishes but is instead, like most qualitative methods, more akin to a craft that requires considerable skill and practice. That said, we believe the method draws sufficiently on existing theoretical and methodological frameworks that other competent qualitative researchers should be able to use it within other behavioural domains. We would argue that this is important and necessary given the potential value in gaining greater knowledge about how we might more effectively deploy affect and think through ideology within public health interventions designed to effect behavioural change.

## Conclusion

This paper contributes to the field through delivering a worked example of how affect and ideology can be traced within intervention materials and how these frame an array of assumptions underpinning the development and deployment of intervention materials with specific populations. It focuses on connecting the distal, cultural determinants of behaviour change with key cultural signs and symbols. Within this worked example, we show how particular signs and symbols tend to be employed within intervention materials and we illustrate how population reach and engagement in this particular field seems to collude with potentially problematic and particularly constraining signs (e.g., porno-chic) whilst also largely neglecting established affective motivators such as fear and threat. In relation to the future intervention development, or the commissioning of similar interventions, our analysis suggests that particular attention should be paid to critically reflecting on this reliance upon a single set of cultural signifiers. Furthermore, intervention development and commissioning briefs should marry the insight of the distal determinants of behaviour with those concerned with the more proximal determinants (Langdridge et al., 2019; McDaid et al., 2019) in order to maximize the possibility of effecting behavioural change.

## Figures and Tables

**Table 1:** Interventions materials available for analysis

Intervention	Number of materials
Brady et al. (2014) – England, UK	3
Check it out (Guy et al., 2009) – Victoria, Australia	2
Crowdsourcing video (Tang et al, 2016) – China	1
Drama downunder (Pedrana et al., 2012; Wilkinson et al., 2016) – Victoria, Australia	22
Get Tested with Via Libre (Blas et al., 2010) – Lima, Peru	2
Gimmie 5 min (McOwan et al., 2002) – England, UK	4
Health Marketing intervention video (Tang et al, 2016) – China	1
Hottest at the start (Gilbert et al., 2013) – British Columbia, Canada	6
I’m testing (James, 2015) – England, UK	16
Make your position clear (Flowers et al., 2013) – Glasgow, Scotland	6
POCT (West et al., 2015) – England, UK	1
Talking about HIV (Hirshfield et al., 2012) – United States	1
The morning after (Chiasson et al., 2009; Hirshfield et al., 2012) -United States^a^	2
United against AIDS (Prati et al., 2016) – Italy	2
Count me in (Hickson et al, 2015) – England, UK	0
Erausquin et al. (2009) – Los Angeles County, USA	0
HIV wake up intervention (Hilliam et al, 2011) – Scotland, UK	0
I did it (Hickson et al., 2015) – England, UK	0
What are you waiting for (Gilbert et al, 2013) – British Columbia, Canada	0
You know different (Thackeray et al., 2011) – United States	0
Tu Amigo Pepe (Solorio et al., 2016) – Seattle, USA	0
Total	69

a Three films included in systematic review but not available for this study due to dead links, only 2 still images found.

**Table 2:** Coding for affect and ideology

Intervention	Intervention materials description	Coding of affect and ideology
Brady et *al.* (2014)	Three posters, contrasting in style: Poster 1 is empty double bed with ruffled sheets and pillows as if there has been sex. This is stamped with a red ‘warning’ message with the text ‘HIV test result expired here’ and separately ‘Update your status’ in which threat is evoked, the threat of an ‘expired’ text result implying risk of infection. Poster 2 is text only albeit with some coloured and bolded. The word ‘HIV’ is embedded within ‘Think’ and ‘Without Knowing’ in red. Poster 3 is a close-up front facing headshot of an attractive, young, white, smiling man looking directly at the viewer. It includes the words ‘I ordered my test online’ with text nearby stating, ‘It starts with me’.	Sexual desire and ideology of ‘pornographication’: Use of ruffled sheets on bed although no people. Classic cinematic trope of desire/romance. The bed also serves to symbolize romance and intimacy, not simply desire.
		Fear, threat and regret: ‘expired’ implies the threat of death Red text evokes threat (in Western context of intervention). ‘Taken a risk’ and notion of ‘Update your status’ evoke regret.
		Fear, threat, and regret: Invoked through the use of red and bold text, ‘HIV without knowing’ all in red interspersed within black text. Only text.
		Sexual desire and identification: The figure is inviting the viewer to join him in stopping HIV (eye gaze to viewer and smile with words We can stop HIV’). His own behaviour is signalled ‘1 ordered my test online’ inviting identification.
Check it out (Guy *et al*., 2009)	Two posters with very different imagery. Both posters are impersonal and somewhat abstract but with an attempt at humour by association (cock/cockerel and dropping pants): Poster 1 consists of an image of two cockerels face to face with the text above ‘Let’s talk man to man’. Poster 2 consists of a man’s naked legs and dropped trousers from the knee down. This is overlaid with the text ‘Another excuse to drop your pants’.	Sexual desire and humour: Rather abstract with an attempt at humour through the ‘cock’ implication. Masculine ideology Invoked through use of cockerel images and wording ’Let’s talk man to man’. Notions of male agency, frankness, openness and honesty implied.
		Sexual desire and humour: Abstract identification through humour, dropping trousers for sex akin to dropping trousers for a test.
Crowdsourcing video (Tang *et al*., 2016)	Video revolves around a classic ‘falling in love’ narrative style in which two young attractive (Chinese) men first see each other by chance, form a relationship and then explore the need for testing together as a couple. The film is softly lit and accompanied by gentle piano music. Whilst set in standard public context there is considerable same-sex/gay symbolism including same-sex posters and rainbow flags.	Romance, love and identification: Two figures, not engaged with viewer (only each other). Strong narrative style drawing on contemporary (heterosexual) cinematic romance ideology (chance meeting, stolen glances etc). Uses notion of loving one’s partner as the affective means for mobilizing action within a couple (taking responsibility for looking after the other). Strongly focused on couple as active agent. Identification key albeit this is in large part through a rather heteronormative relational structure.
Drama downunder (Pedrana *et al*., 2012; Wilkinson *et al*., 2016)	A large series of poster images in a variety of categories although all with the same singular (white) male figure (mostly in his underpants), using the same visual language. They are consistent in form, albeit evoking somewhat different affects, depending on the context: Drama series involve the smiling/laughing naked torso of the male figure overprinted with a cockatoo (or kitten or fish or puppy) and the phrase ‘feeling cocky?’ or ‘scaredy-cat?’ or ‘anything fishy?’ or ‘play ball?’. Series focused on the seasons involving the same male figure in different seasonal settings. The final set of images feature the same model mostly again in white underpants with a series of images and overprinted phrases.	Sexual desire and ideology of’pornographication’, mediated by humour: Images use an image of an attractive naked (white) man alongside humour (and cute animals) to evoke a sexually playful affect with the viewer. Sexual identification (with the naked smiling figure), mediated through use of humour. Humour acts to ameliorate the sexual in this imagery (less ‘pornographic’ in tone).
		Sexual desire and ideology of’pornographication’: Autumn (with amber fallen leaves) is highly sexual with naked smiling figure, shot from overhead, laying in leaves. The image is evocative of other images in the public domain associated with sexuality and romance. Spring and summer are less sexual and more romantic, albeit similar in style. In these images, the model’s pants are visible (‘tighty whities’) whilst in the first his complete nudity is implied through the positioning of leaves. Winter involves the affective mood shifting from sexual/romantic to humour, with the model in his pants shivering and pulling a mock unhappy face in the cold snowy weather.
		Sexual desire and ideology of ‘pornographication’, mediated by humour: Most deploy humour (‘its not long or hard’) such as sexual innuendo although one – perhaps unintentionally – may evoke fear. This image is an image of the model with his body on fire and the phrase ‘don’t let things get out of control’. It is clear that his frightened expression is intentionally faked but even still the sense of a person engulfed in flames is potentially powerfully threatening and fear invoking.
Get Tested with Via Libre (Blas *et al*., 2010)	Two videos, different in content and style but all based in Peru, which frames ideological context for all material: Video 1: Strong narrative component at the heart of this video in which the young male character (looks like a teenager) plays out a scene in which he has got drunk and had unprotected sex with a ‘known fag’. Following rumour, he then fears being infected and so must get tested. The central character then addresses the audience directly in a happy smiling manner upon receiving a negative HIV test. Video 2: This video also has a strong narrative component telling the story of a young man discussing his fears of contracting HIV and belief that it is probably better not to know. He is persuaded by his friend to go and get tested. He is seen to be relieved at his ‘negative’ diagnoses. At this point the narrative is undermined by his friend declaring his positive diagnosis and that he is ok (cannot be read as positive) and is receiving treatment at the clinic. This character then addresses the audience positively at the end.	Fear, threat, and regret: Fear, threat, and regret primary affects, revolving around character identification. Strong narrative element (clear story) focused on fear that is only resolved affectively upon taking the test whereupon his demeanour changes (now smiling and looking directly at camera). Film evokes a heavy sense of regret and then relief upon being tested (negative).
		Problematic identities: The material is ideologically complex and overladen with a sense that a gay identity is negative (character in Video 1 refuses that identity distinguishing himself and his behaviours from that of a ‘fag’). Film operates with a sense of an HIV identity being problematic (hence the relief as a ‘negative’ test result).
		Fear, threat, and regret: In a similar manner to Video 1, fear is very present in the principal character (fear of having HIV). Regret is mobilized through the friend character persuading the principal character to find out one way or the other as it is best to know. Relief is shown clearly upon receiving a negative test result.
		Problematic identities: HIV is normalized to some extent by the other character (the older friend) who declares his own positive status (following principal character receiving a negative result). But even here the notion of ‘passing’ is presented.
Gimmie 5 min (McOwan *et al*., 2002)	A series of images of naked (face and torso only) men alongside the phrase ‘Gimmie 5 min, and then quite a large amount of detailed text. The images feature normatively beautiful and fit/well-muscled young men from a variety of ethnic backgrounds all looking direct to camera in a classic seductive model pose (the ‘smize’).	Sexual desire and ideology of’pornographication’: Sexual desire is centrally figured, drawing the viewer in (to an information resource) through engagement with a visually beautiful erotic form. Men once again not reflective of men more generally but clearly models designed to attract (to give the posters ‘5 min). Models beauty also signals health and well-being. None deviated from an ideal lean ‘fit’ young type.
Health marketing intervention video (Tang *etal*, 2016)	Brightly coloured cartoon video set to the Rondo Alla Turca (Turkish March) from Mozart’s piano sonata no. 11 (K. 311). It consists of a young man under a condom-like transparent umbrella. HIV virus images float in the air as the character walks down the street under the umbrella to the music. He passes a man kissing two women (with the subtitle of ‘Risky sexual behaviours’ appearing), then two men arm in arm (with the subtitle of ‘MSM behaviours’ appearing), and finally a man looking like the quintessential image of a ‘hippie’ smoking a joint (with the subtitle of ‘Drug users’ appearing). There is then a comedic representation of a woman seducing him with him joining her in a hotel whilst dropping his umbrella. He is then seen to search the internet, some dark clouds, and a narrative of him heading for testing then follows along with information about transmission and testing. He tests negative and leaves happy with a ‘thumbs up’ given.	Sexual desire, mediated by humour: Central narrative is founded on desire, albeit little sense of pronographication, with the given example of a man being seduced into having sex in a hotel with what appears to be a random woman. Whilst there are exemplars of activity including MSM named the primary narrative is heterosexual. Adult cartoon quality implies humorous content. Exploding love heart eyes for example are resonant of typical cartoon comic devices and the entire plot is light-hearted in affective tone. The music further supports the light comic style. The brief appearance of dark clouds at the possibility of infection engages a shift in affective tone but this is a very brief move away from the general gently comic affective tone.
		Fear, threat, and regret There is a sense of fear and regret implied here with the failure to carry the umbrella when seduced and black clouds hanging over the character when seeking out information about testing.
Hottest at the start (Gilbert *et al*., 2013)	Three primary images repeated in poster and postcard versions. The images depict sexual encounters between two men in three different settings (lift, kitchen, gym locker room). The men are partially clothed in one image and completely naked in the other two.	Sexual desire and ideology of’pornographication’: Images are strongly sexual and speak to a gay male pornographic aesthetic, with all three inferring anal sex although no genitals are visible. −Choice of locations (lift, kitchen, and locker room) is classic within romantic and pornographic narratives. Men are handsome, well-muscled, with trimmed body hair, the stuff of pornographic fantasy. None deviate from being lean and fit. Wording about ‘HIV being a powerhouse in the sack’ sits as a warning against the notion of these men being ‘powerhouses’ in sex. Affective tone is deeply erotic, drawing the reader in through a practised gay erotic gaze (a voyeuristic mode of engagement).
I’m testing (James, 2015)	A consistent series of posters featuring some potentially recognizable gay public figures (e.g., ‘Dr Christian’) with 3 men and 1 woman. The Dr image is further enhanced with him wearing a stethoscope (implying trust: If a doctor tests, then so should 1). The images are primarily the upper clothed torso of an attractive smiling person wearing an ‘I’m testing’ bright orange t-shift set against a vivid blue background. There is also the web address ‘startswithme.org’ featured prominently.	Flattened affect: Colourful, sunny (bright oranges and vivid blues) and smiling images with a sense of warmth and contentment at engaging in this practice. Evoke a (positive) clinical sensibility.
Make your position clear (Flowers, McDaid, *et al*., 2013)	A series of images with the same two men featuring throughout. Both are likely to be classed as attractive albeit not quite as extremely muscled as some other sets of intervention materials (e.g., Gilbert et al.). They are clothed in two images and naked in the others, albeit without genitals showing. The two clothed images reflect two men in ostensibly ordinary settings (supermarket and office). However, both are dressed well, notably in the office, implying middle-class professionals. In the naked images they are engaged in different sexual positions with references to public discourses about often sexual ‘positions’, then associated with the phrase ‘whatever position you are in, sexual check-ups have a part to play’. There is also an image of an open packet of condoms and lube in the corner functioning as an additional supplementary message to the primary one.	Sexual desire, ideology of’pornographication’, mediated by love and romance: Men in the images are focused on each other (not the viewer) and give the impression of being in a loving relationship. Reader is drawn in through both a romantic aesthetic (meeting at the watercooler or in the supermarket: the ‘everyday’) and a sexual aesthetic (via the explicit visual imagery, albeit through images that also appear loving). Naked images mostly lack a context in contrast to the clothed images, presented against a blank background. Affective mood is both romantic and sexual/erotic.
POCT (West *et al*., 2015)	Male (doctor/nurse) is shown testing a middle-age white woman, talking her through the process.	Flattened affect: Realistic portrayal with little attempt to do anything other than demonstrate the everyday practice of testing in a medical office. No attempt to engage affect beyond potential for some anxiety reduction through knowledge.
Talking about HIV (Hirshfield *et al*., 2012)	Video not available due to dead link (www.hivbigdeal.org –deadlink)	n/a
The morning after (Chiasson *et al*., 2009; Hirshfield *et al*., 2012)	Video not available due to dead link (www.hivbigdeal.org –deadlink) Two images of manhunter ads show usual manhunter imagery of naked torso man advertising the ‘morning after’ video.	Sexual desire and ideology of’pornographication’: Limited data but similar to other campaigns based on sexual desire using a gay porn aesthetic.
Tu Amigo Pepe (Solorio *et al*., 2016)	Not possible to analyse (insufficient content) given only screen shots of website available.	n/a
United against AIDS (Prati *et al*., 2016)	People of different ages naked albeit tastefully hidden behind a large red ribbon. The people are in black and white with the red ribbon around them and on the floor in colour.	Flattened affect: Presence of men and women of all ages desexualized the imagery, with it looking akin to some sort of political protest or artwork. Image is aesthetically pleasing but not using affect beyond an artistic visual appeal to engage the viewer.

## Data Availability

Research data are not shared.
